# *In Vitro* and *In Vivo* ^1^H-MR Spectroscopic Examination of the Renal Cell Carcinoma

**Published:** 2012-06

**Authors:** F. Süllentrop, J. Hahn, D. Moka

**Affiliations:** *Institute of Inorganic Chemistry and the Department of Nuclear Medicine University of Cologne, Germany*

**Keywords:** renal cell cancer, lipid metabolism, ^1^H-magnetic resonance spectroscopy

## Abstract

**Background:**

Paraneoplastic effects are some of the major side effects of advanced renal cell carcinoma (RCC). Magnetic resonance spectroscopy (MRS) is known as a powerful tool to study cancer cell metabolism and cancer cell – host interactions. Aim of this study was to assess tumor cell metabolism and systemic effects using ^1^H-MRS.

**Methods:**

Spectroscopic analysis of 10 patients with RCC was compared with those of 15 healthy volunteers. Local tumor metabolism was assessed using image-guided ^1^H-*in-vivo*-spectroscopy in a 1.5 Tesla MR whole body tomograph. Systemic effects of RCC were measured using ^1^H-High-Resolution (HR) spectra of blood plasma samples in a 500 MHz Bruker DRX 500 spectrometer.

**Results:**

*In-vivo*-spectroscopy can significantly differentiate tumor tissue from healthy renal tissue by comparing their lipid composition. Moreover after detailed assignment of the various metabolites in blood plasma in the *in-vitro*-HR-spectra significant systemic alterations could be identified in patients with RCC especially regarding lipid and amino acid metabolism.

**Conclusion:**

This work indicates that using ^1^H-MRS both changes in tumor metabolism and resulting systemic/paraneoplastic effects can be assessed in patients with RCC. This approach therefore offers scope for diagnosis and therapy evaluation.

## INTRODUCTION

Due to more common use of ultrasound and computer tomography as well as magnetic resonance tomography as means of medical examination today the diagnostics of the renal cell carcinoma (RCC) has changed decisively in the last years.

Moreover RCC has various kinds of local metabolic alterations and of so called paraneoplastic syndromes, which are mainly caused by endocrine activities of advanced renal tumors (1).

The changes in the lipid and amino acid metabolism observed in renal cell carcinoma are the subject of this paper. Pathobiochemical changes in tumor metabolism can be observed locally in the tumor itself (2) or peripheral in blood plasma of patients with RCC (3). The clinical interest in the examination of changes in metabolism has increased with many studies demonstrating the strong influence of tumor tissue on lipid and phospholipid metabolism (4-7). Alterations in lipid metabolism in RCC tissue have been documented by various investigators. Karlsson *et al*. found higher concentrations of gangliosides and a different pattern of ceramides in renal carcinoma tissue in comparison to normal renal tissue (8), while an abnormal cholesterol metabolism in renal clear cell carcinoma was described by Gebhard *et al*. Clear cell cancer tissue contained more total cholesterol and more esterified cholesterol than was found in normal kidney (9). Clayman *et al*. reported a decrease in LDL-receptor activity but an increase in the rate of cholesterol synthesis in malignantly transformed renal tissue (10). Reduced LDL-receptor mRNA levels in human renal cell carcinoma tissue in comparison with normal kidney tissue were observed by Rudling *et al*. (11). An increased synthesis of sulfolipids due to elevated glycolipid sulfotransferase activity could be detected in human renal cell carcinoma tissue (12, 13). Serum of renal cell carcinoma patients also showed an increased activity of glycolipid sulfotransferase compared to that of normal controls and patients with other urological tumors (14). Renal tissue from Syrian hamsters with primary kidney tumors revealed lower levels of phospholipids compared to those found in kidneys from normal hamsters. Furthermore, the phospholipid composition of the tumors differed from that of normal kidneys, meaning an increased percentages of PC and decreased percentages of SM being found (15).

Previously we investigated tissue samples of RCC using a combination of the high resolution magic-angle spinning method and the pattern recognition method. We detect marked alterations in different lipid components mainly originating from triglycerides and cholesteryl lipids in the cancer tissue (2, 16, 17).

In another previous publication we already found systemic effect in patients with RCC by changes in phospholipid concentrations in blood (3). Other investigators found systemic changes in lipids of blood plasma in patients with thyroid cancer (18), hematological cancers (19-21) or digestive tract tumors (22).

The aim of the present investigation was to evaluate the systemic alterations in concentrations of lipids in plasma using ^1^H MR spectroscopy and to correlate the changes with the changes in lipids in the tumor itself by using the *in vivo* spectroscopy.

## MATERIALS AND METHODS

### Materials

D_2_O was obtained from Deutero GmbH (Herresbach, Germany), all other chemicals were purchased from Sigma-Aldrich Chemie GmbH (Deisenhofen, Germany).

### Subjects and Samples

Ten patients with firmed advanced RCC (5 male, 5 female, the average age was 65 years) and 15 healthy volunteers (7 male, 8 female, the average age was 40 years) were included in the study after detailed clinical questioning. None of the patients with RCC showed renal dysfunction (determined using dynamic renal scintigraphy (23)), cachexia or any disease that could lead to a disturbance of lipid metabolism. The study was approved by the Hospital Human Rights Committee (Institutional Review Board) and written informed consent was obtained from all patients.

The two-sided unpaired Student’s t-Test was used to establish the statistical significance of the results. *P* values ≤0.05 were considered to be significant.

Plasma samples for the *in vitro* ^1^H MR spectroscopic investigations were obtained preoperatively and prepared as follows:

Blood samples (10 ml) were collected in sterile EDTA-containing tubes after a 12 h fast. The plasma was separated by centrifugation (3000 g for 10 min.). When hemolysis occurred, the sample was discarded. The plasma was stored at -80°C until required for measurements. The standard solution for the ^1^H MR spectroscopic measurements contained 25.29 mmol/l of tetradeuterotrimethylsilylpropionate as an internal reference compound for quantification purpose, D_2_O as a solvent and MR lock compound in 25 ml solution.

### MR spectroscopy

***In vitro*^1^H MR spectroscopy.** The one-dimensional 500 MHz (11.7 Tesla) ^1^H MR spectra of the blood plasma were recorded on a Bruker DRX 500 spectrometer, sample temperature being held at 310 K, using a 5 mm inverse probe head. The spectra were recorded using a pulse sequence that suppresses the water signal by means of presaturation (Bruker© program *noesypr1d*) which was found to be the best method for suppression of the water signal. The SFO1 (standard frequency for measurement) was set on the water signal. Other acquisition parameters were as follows: pulse length 12.0 μs (90°), sweep width 6009 Hz, time domain 32 k data points, number of scans 256 and repetition time 5.7 s. Longitudinal ^1^H relaxation times (T_1_) of the reference compound and the lipids contained in standard plasma samples under standard conditions were determined using an inversion recovery pulse sequence (28). The resulting T1 values were 0.6 s for the reference compound and 0.3-0.4 s for the signals of the lipids. The spectra were processed using X-WIN-MR 2.0^®^ Bruker Analytische Meßtechnik GmbH. Chemical shift assignments were referenced relative to the reference compound at 0 ppm. The peak areas were determined by iterative deconvolution using a program of the PERCH project (Kupio, Finland) (24).

***In vivo*^1^H MR spectroscopy.** MR tomograms (T1 weighted, resting expiratory position) were used to identify healthy kidney tissue and kidney tumor tissue to define the volume of interest (VOI) for the *in vivo* spectroscopy. The detection of the tomograms was carried out by using a ring coil with a diameter of 18 cm that was also used for the spectroscopy. The patient’s position was supine on the ring coil, so that the distance between the coil and the VOI was as small as possible (20 × 20 × 20 mm; distance coil to tumor between 5.6 and 8.1 cm). All measurements were carried out in a 1.5 T whole body tomograph (Gyroscan ACS, Phillips, Best, Netherlands).

The acquisition parameters were as follows: The one-dimensional 63.89 MHz (1.5 Tesla) ^1^H MR spectra of the kidneys were recorded using a pulse sequence that suppresses the water signal by means of excitation. This method had turned out to be the best method for suppression of the water signal. The SFO1 (standard frequency for measurement) was set on the water signal. Other acquisition parameters were as follows: sweep width 2000 Hz, time domain 1 k data points, number of scans 256, repetition time 2 s and echo time 25 ms (spectroscopy acquisition time: 9 min.). The spectra were processed and the peak areas were determined by iterative deconvolution using WIN-MR 5.1^®^ Bruker Analytische Meßtechnik GmbH.

### Quantitative evaluation of the ^1^H MR

Due to the overlap of MR signals, for the integration of the ^1^H MR spectra of blood plasma (*in vitro*) and tissue (*in vivo*) the use of deconvolution methods is mandatory. Both programs used are described briefly in the next paragraphs.

**Deconvolution using the program PERCH (*in vitro* spectroscopy).** The program PERCH (24) offers the possibility of a far-reaching automatic deconvolution of MR spectra. In this context the spectrum is tested half automatically on the shape of the signal and the level of the noise. The fixing of the level of noise makes an automatic selection of signals possible. Afterwards the signals are simulated by PERCH. PERCH fits the shape of the signals iteratively to those signals obtained experimentally. The parameters height, width and frequency as well as (if necessary) the baseline are optimized to get a minimal rest integral in the difference spectrum. Because of the high grade of automation this method can be used even if the spectra show a large number of separate signals. However, for the deconvolution of big broad next to small sharp signals this method doesn’t lead to a significant result in all cases. In such cases, the rest signal of the broad MR signal may overlap the small sharp signals that could be seen in the spectrum themselves. This consequently leads to a not tolerable misalignment for the integration of the small signals. This is a general problem of iterative integration’s, carried out with a computer.

However, in the ^1^H MR spectra of blood plasma the case discussed above may take place, but the resulting problem can be avoided. If the spectrum is evaluated in parts being smaller than the underlying broad MR signal the broad MR signal can bee seen (without making a big mistake) like a deformation of the baseline of the spectrum. Moreover, the program PERCH offers the option of an additional automatic baseline correction.

**Figure 1:**
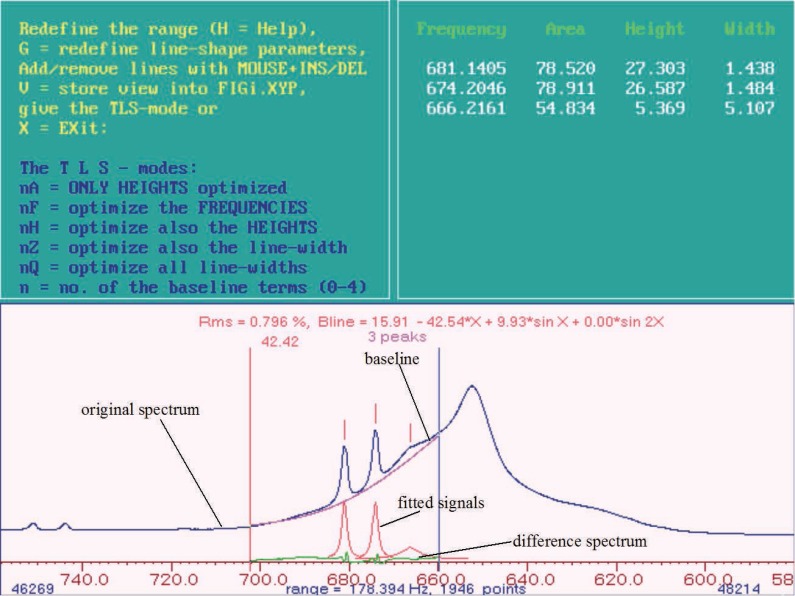
^1^H MR Spectrum of blood plasma (only the part with the signal for CH_2_ groups of lipids (big signal) and signals for lactate can be seen here). First step of the line shape fitting, fitting for the signals of lactate.

**Figure 2:**
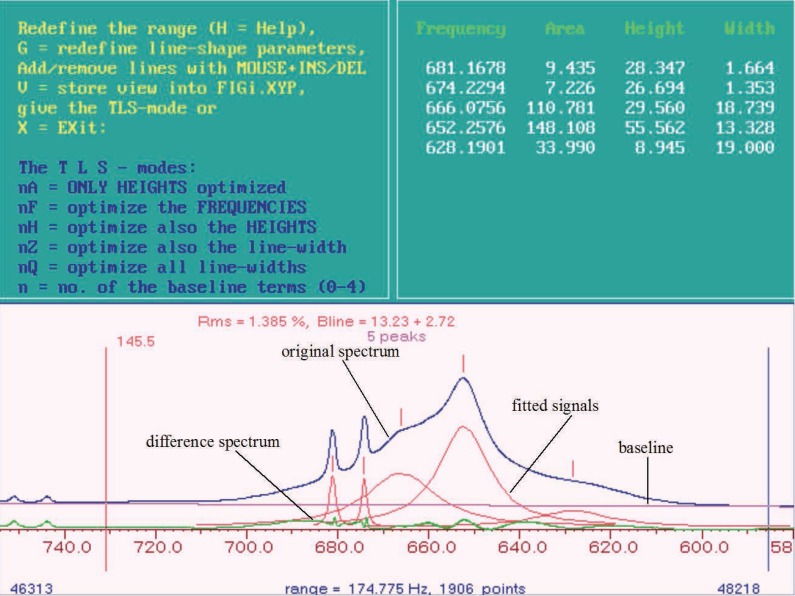
^1^H MR Spectrum of blood plasma: line shape fitting second step, fitting of the big signal.

**Figure 3:**
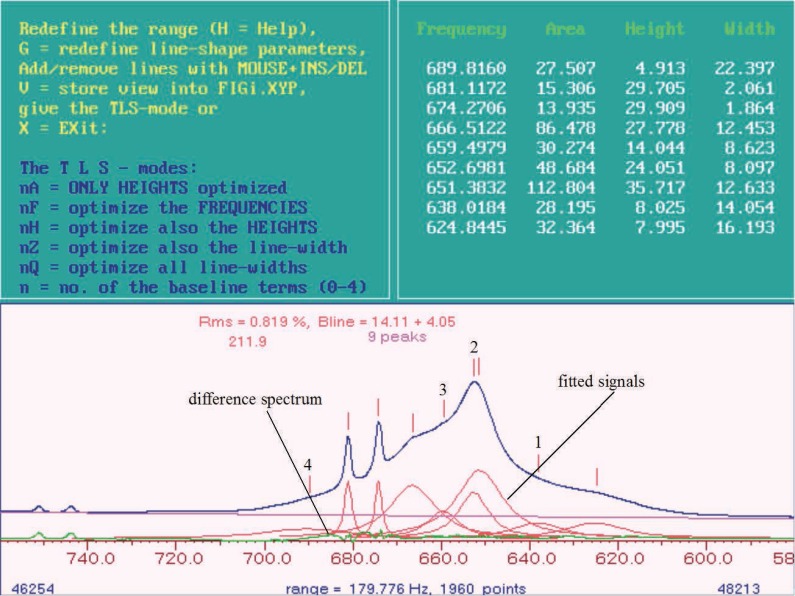
^1^H MR Spectrum of blood plasma: line shape fitting, general view. The model signals that were put in additionally are marked by the numbers 1-4.

For a better understanding of the structure of PERCH the procedure of the integration and handling of the program is illustrated by an example. The Figures 1-3 show step by step the integration of three signals from a ^1^H MR spectrum of blood plasma using PERCH. The biggest signal is caused mainly through CH_2_-groups of fatty acids which can be assigned to LDL and VLDL but it also contains CH_2_-groups of fatty acids that can be assigned to HDL and free fatty acids. The small signals can be assigned to lactate. Table 1 gives an overview of all signals that had been integrated in this investigation using PERCH. All of these signals had been fitted in the same manner as shown for the three signals in Figure 1.

**Table 1:** Values for the parameter G

peak	assignment	limits for the integration and chemical shift values (given in Hz)		values for G	
				line width in Hz	Gaussian part in %

1	reference substance	-100 – 150	0	8^b^	10^b^
2	CH_3_ group of cholesterol in HDL (C18)	300 – 380	345	7	5
3	LDL1→ CH_3_(CH_2_)_n_	380 – 560	420	5	5
	VLDL1→ CH_3_CH_2_CH_2_HC=		435	additional peaks	
	terminal CH_3_ groups of fatty acids			4	4
4^a^	Valin –CH_3_	490 – 560	500	0.5	5
			530		
5	LDL2→ (CH_2_)_n_	580 – 760	625	5	5
	VLDL2→ CH_2_CH_2_COOR		645	additional peaks	
	CH_2_ groups of fatty acids		660		4
	originating from HDL and free fatty acids			4	
6^a^	lactate –CH_3_	660 – 690	675	0.5	5
7^a^	alanine –CH_3_	720 – 760	745	0.5	5
8	-CH_2_CH_2_COOR groups originating from fatty acids	760 – 840	770	5	5
9	-CH_2_CH_2_HC=CH- groups originating from fatty acids	840 – 900	870	5	5
				additional peaks	
				2	2
10	-CH_2_HC=CH- groups originating from fatty acids	950 – 1080	1015	5	10
11^a^	-NHCOCH_3_- both signals refer to	1020 – 1080	1030	2	10
12^a^	composite acetyl signals of a α_1_-acid glycoprotein		1050	1.5	10
13	-CH_2_COOR groups originating from fatty acids	1080 – 1180	1125	5	5
				additional peaks	
				3	3
14	-HC=CHCH_2_HC=CH- groups	1340 – 1435	1380	7	7
	originating from fatty acids			additional peaks	
				3	3
15	ε-CH_2_-, originating from amino acids in albumin	1435 – 1540	1445	2	5
			1490		
			1520		
16	-N^+^(CH_3_)_3_ groups of different, choline containing molecules in lipoproteins	1605 – 1675	1625	5	10
				additional peaks	
				5	5
17^a^	H2, β-glucose	1625 – 1675	1635	1	5
18^a^	-CH_2_, free glycerol	1825 – 1860	1835	0.5	5
19	=CHCOOR, glyceryl of lipids (fatty acids)	2570 – 2625	2601	2	5
20^a^	anomeric H1, α-glucose	2625 – 2640	2631	0,5	5
21	CH groups of unsaturated fatty acids in lipids -CH=CHCH_2_CH=CH-and =CHCH_2_CH_2_-	2625 - 2780	2656	10	10
				additional peaks	
				5	10

The line widths of the signals are given in Hz and Gaussian parts in per cent for each signal. The assignments are taken from literature (24, 25).

a The signals marked with this symbol have been integrated separately (and their values stored in a different “top” data file) because of their intensity being so weak that they could not be fitted together with the signals for the lipids;

b The values for the reference signals are determined for each sample in the part of the program named “PIC” (see above).

However, before the signals are fitted the reference signal must be fitted first. The starting values for the fitting of the reference signal and the other signals in the spectra are generated in the part of the program named “PIC”. The reference signal is fitted in the same way as described for the big signal as well as the small signals in this example here. For the analysis of the spectra in this thesis a one model signal was sufficient for the fitting of the reference signal but if necessary more than one model signal has to be used for the integration.

For the fitting of the small signals the positions of the lines of the model signals are set at first using the cursor (that means two lines are inserted in the original spectrum see Figure 1). The positions of the lines are listed in Table 1. To get good starting parameters for the fitting of the signals a baseline correction within the region for the integration using the parameters 1Q and 2Q is made. Afterwards the height, frequency and line width of the signal which is to be integrated are optimized simultaneously using the parameters A, F, H, Z, Q. The parameter G (Table 1) is used to determine the line width and the Gaussian part of the signal (except for the reference signal). Finally the signal is fitted again using the parameter Q (which only affects the line width of the signal). With the commands 1Q and 2Q (if necessary 3Q) a baseline correction is performed again within the region for the integration (Table 1). The black line in Figure 1 marks the original spectrum, which was prepared only by making a zero order phase correction (no corrective first order phase fault) and a first order baseline correction within the region from –3 to 10 ppm in the spectrum. The small grey line under the black line in Figure 1 corresponds to the signals which were fitted as described above. Furthermore the optimized baseline can be seen in this figure as well as the rest signal of the difference spectrum, which results from the difference between the original and the fitted spectrum. In this first round of fitting the signal the small signals as well as the reference signal are integrated alone. The results are stored in a so called “top” data set.

In the second round the big signal and the reference signal are now integrated. The small signals are fitted again, together with the big signal. In Figure 1 there are two signals for lactate and one big signal which can be assigned to mainly CH_2_ groups of fatty acids, belonging to LDL and VLDL. However, CH_2_ groups of fatty acids belonging to HDL and to free fatty acids are involved. The procedure is the same as described above for the lactate signals. First of all model signals are put in the signal. The signal consists of different compounds (see above) which show different chemical shifts but in these spectra only one signal can be seen. So the model signals for the different compounds (of which the assignments are known see Table 1 and (25)) are put in the signal using the cursor. In this example three signals are known that can be used as model signals: one for CH_2_-groups originating from LDL with a chemical shift of 625 Hz, one for CH_2_-groups of fatty acids from VLDL with a chemical shift of 645 Hz and one which can be assigned to CH_2_-groups originating from other fatty acids, for example HDL, with a chemical shift of 660 Hz. These chemical shift values are the starting values for the fitting of the signals, the frequencies are released and held on to a fixed frequency. Afterwards the procedure is the same as described for the lactate signals, which are fitted simultaneously here. The result of this procedure can be seen in Figure 2. The figure shows the original spectrum, the fitted signals, the baseline and the difference spectrum for the part of the ^1^H MR spectrum of blood plasma used in this example.

When the difference spectrum is a nearly flat line, the integration is finished and the values are stored in a so called “top” data file again. The values for the integration of the small signals are taken from the first “top” data file (see first step of the line shape fitting). This procedure should be done, like explained before, because of the difficulties for the fitting of small sharp next to big broad signals.

If, as seen in this example (Figure 2), only positive aberrations to a flat line can be seen in the difference spectrum. Further model signals are added to areas showing positive aberrations. For this example this means four additional model signals. Using the parameter G the line width a Gaussian part of for all of the signals is determined anew (Table 1). Using Q a new optimization of the line width of the signals and with 1Q and 2 Q a new baseline correction is carried out within the given limits for the integration. Further model signals are inserted like described here until the difference between the real and the fitted signals are nearly zero, which shows in the difference spectrum consists of a nearly flat line. The result is displayed in Figure 3. The sum of all areas of all model signals (seven in this example) leads to the value for the big broad signal, which should be fitted in this example.

**Figure 4:**
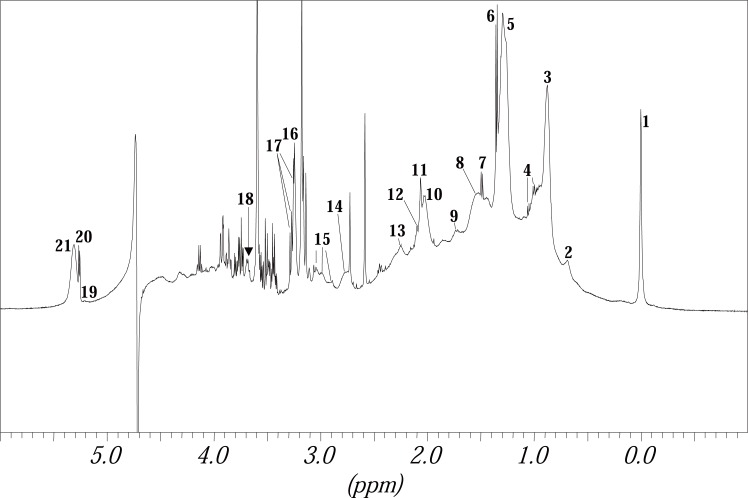
^1^H MR Spectrum of blood plasma of a healthy volunteer.

For a better understanding as the spectra is quite complicated, Figure 4 shows a ^1^H MR spectrum of blood plasma of a healthy volunteer as a comparison. The peak assignments and the chemical shifts are given in Table 2. The spectra for patients with RCC do not differ from those of healthy volunteers concerning the peak assignment. The difference only shows in the areas of the peaks being proportional to the concentration of the substances that belong to the peak.

**Table 2:** Chemical shifts (in ppm), multiplicity (taken from literature (24, 25)) and assignment of the peaks investigated in this paper

Peak	Chemical shift (ppm)	Multiplicity	Assignment

1	0	s	reference substance
2	0.69	m	CH_3_ group of cholesterol in HDL (C18)
3	0.88	t	LDL1→ CH_3_(CH_2_)n
			VLDL1→ CH_3_CH_2_CH_2_HC=
			terminal CH_3_ groups of fatty acids
4	1.00	2 × d	Valin –CH_3_
	1.06		
5	1.28	m	LDL2→ (CH_2_)_n_
			VLDL2→ CH_2_CH_2_COOR
			CH_2_ groups of fatty acids
			originating from HDL and free fatty acids
6	1.35	d	lactate –CH_3_
7	1.49	d	alanine –CH_3_
8	1.54	m	-CH_2_CH_2_COOR groups originating from fatty acids
9	1.74	m	-CH_2_CH_2_HC=CH- groups originating from fatty acids
10	2.03	m	-CH_2_HC=CH- groups originating from fatty acids
11	2.06	s	-NHCOCH_3_- both signals refer to composite acetyl signals of a α_1_-acid
12	2.10	s	glycoprotein
13	2.25	m	-CH_2_COOR groups originating from fatty acids
14	2.76	m	-HC=CHCH_2_HC=CH- groups originating from fatty acids
15	2.89	3 × t	ε-CH_2_-, originating from amino acids in albumin
	2.98		
	3.04		
16	3.25	s	-N+(CH_3_)_3_ groups of different, choline containing molecules in lipoproteins
17	3.27	dd	H2, β-glucose
18	3.67	dd	-CH_2_, free glycerol
19	5,20	m	=CHCOOR, glyceryl of lipids (fatty acids)
20	5.26	d	anomeric H1, α-glucose
21	5.31	m	CH groups of unsaturated fatty acids in lipids -CH=CHCH_2_CH=CH-and =CHCH_2_CH_2_-

The assignment has been done from higher to lower field. Abbreviations: s, singlet; d, duplet; dd, duplet of duplets; t, triplet; m, multiplet, the groups underlined are responsible for the peaks assigned; HDL, high density lipoproteine; LDL, low densitiy lipoproteine; VLDL, very low density lipoproteine.

**Figure 5:**
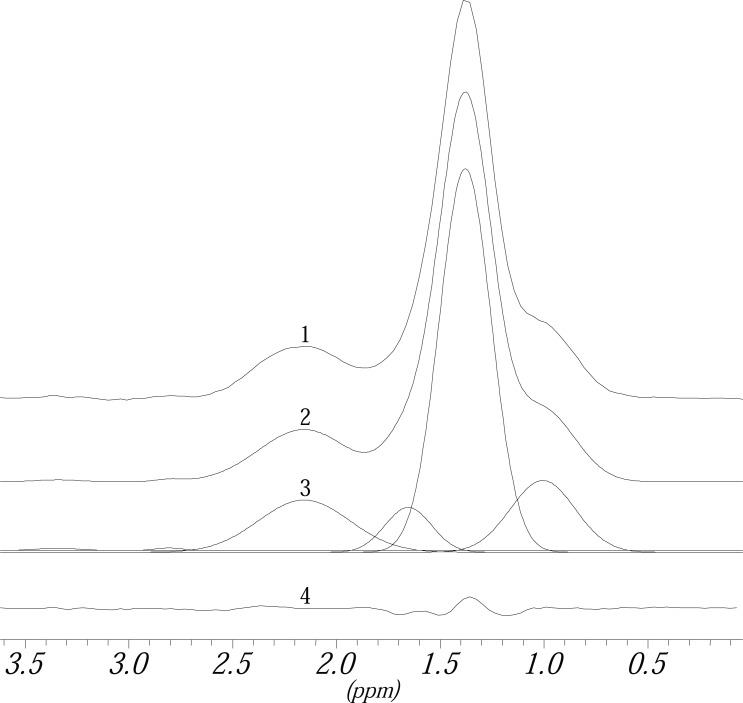
*In vivo* ^1^H MR tumor spectrum of a patient with renal cell carcinoma. As displayed from top to the bottom of the figure: detected spectrum (1), sum of the fitted signals (2), signals displayed separately (3), difference spectrum (4).

**Deconvolution using the program WIN-MR 5.1 (*in vivo* spectroscopy).** The program WIN-MR 5.1 offers the possibility to carry out a half automatically deconvolution. The signals are fitted interactively using a signal shape with a given Gaussian/Lorenzian ratio. The parameter height, line width and frequency of the signals can be varied continuously under optical control. This leads to properly fitted signals and a difference spectrum showing a flat line. Later this fitting can be optimized using the computational iteration. This method is advantageous if there are only few overlapping signals with different line widths in the spectrum. However, there should not be too many signals as the interactive fitting would take too much time for a greater number of signals. The appearance of signals with a variety of line widths is no problem; the “intelligence aided” fitting of the signals avoids the problem of the “local minima” of the computer aided iterations. A typical line shape fitting can be seen in Figure 5.

As described above this program had been used only for the evaluation of the *in-vivo* spectra because the plasma spectra were showing too many overlapping signals and thus interactive fitting would have been too time consuming.

## RESULTS

### *In vitro* ^1^H MR spectroscopy

^1^H MR Spectra had been acquired of five women and five men (average age 65 years) with advanced RCC. The results of the integration of the peaks observed in these spectra (Table 1) were compared to those obtained from a group of 15 healthy volunteers (average age 40 years, 7 male, 8 female).

In Table 3 the results for the means, standard deviations, differences and *p* values obtained from the integration of the peaks of the ^1^H MR spectra of blood plasma are listed for both, patients and volunteers.

**Table 3:** Means, standard deviations (stadev) and *p* values for patients with RCC (RCC) and healthy volunteers (HP)

peak	assignment	RCC	stadev	HP	stadev	diff. %	*p* value

2	CH_3_ group of cholesterol in HDL (C18)	0.0444	0.0234	0.0504	0.0254	-12	0.5687
3	LDL1→ CH_3_(CH_2_)_n_	0.8274	0.3900	0.8930	0.1927	-7	0.5855
	VLDL1→ CH_3_CH_2_CH_2_HC=						
	terminal CH_3_ groups of fatty acids						
4	Valin –CH_3_	0.0082	0.0027	0.0082	0.0017	0	0.9437
5	LDL2→ (CH_2_)_n_	2.1033	0.9163	2.5486	0.9329	-17	0.2669
	VLDL2→ CH_2_CH_2_COOR						
	CH_2_ groups of fatty acids						
	originating from HDL and free fatty acids						
6	lactate –CH_3_	0.2487	0.1567	0.1211	0.0444	105	0.0066
7	alanine –CH_3_	0.0106	0.0038	0.0139	0.0040	-24	0.0678
8	-CH_2_CH_2_COOR groups originating from fatty acids	0.2636	0.1055	0.5913	0.2474	-55	0.0011
9	-CH_2_CH_2_HC=CH- groups originating from fatty acids	0.0441	0.0112	0.0577	0.0139	-24	0.0208
10	-CH_2_HC=CH- groups originating from fatty acids	0.6344	0.2727	0.7623	0.2401	-17	0.2426
11	-NHCOCH_3_- both signals refer to composite acetyl signals of a α1-acid	0.0791	0.0305	0.0621	0.0179	27	0.0980
12	glycoprotein	0.0161	0.0081	0.0117	0.0053	38	0.1790
13	-CH_2_COOR groups originating from fatty acids	0.1766	0.0843	0.2029	0.0733	-13	0.4309
14	-HC=CHCH_2_HC=CH- groups originating from fatty acids	0.1682	0.0668	0.2094	0.0976	-20	0.2773
15	ε-CH_2_-, originating from amino acids in albumin	0.1140	0.0499	0.1545	0.0579	-26	0.0950
16	-N^+^(CH_3_)_3_ groups of different, choline containing molecules in lipoproteins	0.0934	0.0511	0.0832	0.0189	12	0.6904
17	H2, β-glucose	0.1389	0.0788	0.1915	0.0439	-27	0.0466
18	-CH_2_, free glycerol	0.0380	0.0122	0.0420	0.0124	-10	0.4524
19	=CHCOOR, glyceryl of lipids (fatty acids)	0.0078	0.0052	0.0232	0.0111	-66	0.2914
20	anomeric H1, α-glucose	0.0516	0.0201	0.0681	0.0186	-24	0.1828
21	CH groups of unsaturated fatty acids in lipids -CH=CHCH_2_CH=CH-and =CHCH_2_CH_2_-	0.6944	0.2885	0.8772	0.2924	- 21	0.1506

Means and standard deviations are given in mmol/l, referred to the belonging CH_n_ group. If not stated otherwise the peaks refer to components of fatty acids from lipids; significant differences between both groups are marked (*p* value<0.05). RCC, value for patients with RCC; HP, value for healthy volunteers; stadev, standard deviation; diff., difference of the mean values of both groups (in %).

**Figure 6:**
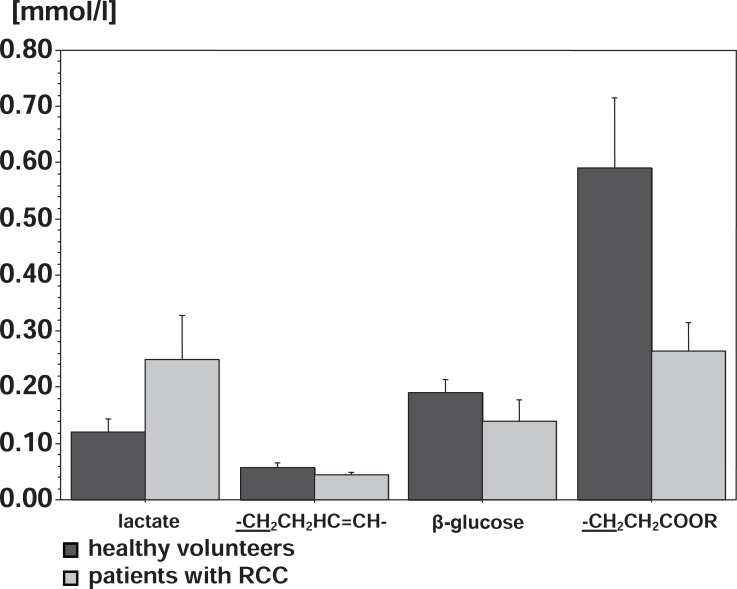
Differences in the concentrations of the substances detected in ^1^H MR spectra of patients with RCC and healthy volunteers.

Figure 6 illustrates the significant differences in the concentrations of the substances detected in ^1^H MR Spectra between patients with RCC and healthy volunteers.

When comparing patients with RCC and healthy volunteers the patients with RCC revealed significant lower concentrations for the components of fatty acids in their blood plasma. Those components include CH_2_ groups of -CH_2_CH_2_COOR and -CH_2_CH_2_HC=CH- (peak 8 and 9). For the other components of fatty acids investigated here and for those that can be assigned to fatty acids originating from VLDL and LDL as well as cholesterol from HDL the patients with RCC also show decreased concentrations in blood plasma. However, these differences are not significant. Moreover, the concentrations of β-glucose and α-glucose are lower for patients, however only results regarding the β-glucose being significant. Other differences between patients and volunteers are given in the lactate and albumin concentration. Lactate being significantly higher and albumin lower (but not significantly lower) in patients. Glucose, lactate and albumin could also be used to check the renal function of the patients. High values of glucose and lactate as well as a lack of albumin would give a hint on a disturbance in the renal function of the patients, a circumstance which would have an influence on the lipid metabolism in the patients. However in good correlation to the renal scintigraphy, despite the higher lactate concentrations no indication on a disturbance in renal function could be found.

The significant alterations of alanine and valine we previously found in renal cell cancerous tissue (3) could not be demonstrated in blood plasma of our patients with RCC.

Table 4 shows the results for different gender in patients with RCC in comparison to the volunteers. Although lacking significance the comparison of the mean values for the concentrations of the various substances from blood plasma leads to following results: Women with RCC show decreased concentrations for all components of fatty acids. The concentrations of glucose and albumin are also lower for female patients. Only the concentration of lactate is higher. The biggest differences between female patients and volunteers can be seen in VLDL2+LDL2, -CH_2_CH_2_COOR and for glyceryl groups of lipids. Contrasting to that, male patients with RCC show no general decrease in concentrations of components of lipids in comparison to male volunteers. The concentrations of components belonging to fatty acids from VLDL1+LDL1, VLDL2+LDL2, and -HC=CHCH_2_HC=CH- groups of fatty acids are even higher for patients than volunteers. The other components of lipids are in the same range for patients and volunteers or are lower for the patients. The biggest (negative) differences can be seen at -CH_2_CH_2_COOR and -CH_2_CH_2_HC=CH-. The concentration of lactate in blood plasma of male patients with RCC is as for female patients, higher than that of the volunteers. However, those of albumin and glucose are lower.

**Table 4:** Mean values for women with RCC (F RCC), healthy women (F), men with RCC (M RCC) and healthy men (M)

peak	assignment	F RCC	F	diff.%	M RCC	M	diff.%

2	CH_3_ group of cholesterol in HDL (C18)	0.0597	0.0651	-8	0.0321	0.0336	-5
3	LDL1→ CH_3_(CH_2_)_n_	0.8017	1.0075	-20	0.8480	0.7622	11
	VLDL1→ CH_3_CH_2_CH_2_HC=						
	terminal CH_3_ groups of fatty acids						
4	Valin –CH_3_	0.0078	0.0083	-7	0.0084	0.0080	5
5	LDL2→ (CH_2_)_n_	2.0578	3.1574	-35	2.1397	1.8528	15
	VLDL2→ CH_2_CH_2_COOR						
	CH_2_ groups of fatty acids						
	originating from HDL and free fatty acids						
6	lactate –CH_3_	0.2211	0.1096	102	0.2708	0.1343	102
7	alanine –CH_3_	0.0086	0.0125	-31	0.0123	0.0162	-24
8	-CH_2_CH_2_COOR groups originating from fatty acids	0.3262	0.6636	-51	0.2135	0.5085	-58
9	-CH_2_CH_2_HC=CH- groups originating from fatty acids	0.0442	0.0577	-23	0.0440	0.0577	-24
10	-CH_2_HC=CH- groups originating from fatty acids	0.6218	0.8739	-29	0.6445	0.6347	2
11	-NHCOCH_3_- both signals refer to composite acetyl signals of a α_1_-acid	0.0976	0.0672	45	0.0643	0.0563	14
12	glycoprotein	0.0228	0.0136	67	0.0108	0.0094	15
13	-CH_2_COOR groups originating from fatty acids	0.1559	0.2128	-27	0.1933	0.1915	1
14	-HC=CHCH_2_HC=CH- groups originating from fatty acids	0.2066	0.2856	-28	0.1375	0.1223	12
15	ε-CH_2_-, originating from amino acids in albumin	0.1331	0.1590	-16	0.0986	0.1494	-34
16	-N^+^(CH_3_)_3_ groups of different, choline containing molecules in lipoproteins	0.0664	0.0885	-25	0.1150	0.0771	49
17	H2, β-glucose	0.1811	0.2087	-13	0.1052	0.1718	-39
18	-CH_2_, free glycerol	0.0332	0.0475	-30	0.0418	0.0333	25
19	=CHCOOR, glyceryl of lipids (fatty acids)	0.0140	0.0479	-71	0.0028	0.0020	42
20	anomeric H1, α-glucose	0.0729	0.0764	-5	0.0346	0.0585	-41
21	CH groups of unsaturated fatty acids in lipids -CH=CHCH_2_CH=CH-and =CHCH_2_CH_2_-	0.7377	1.0624	-31	0.6598	0.6655	-1

The mean values are given in mmol/l referred to the CH_n_ group they belong to. If not stated otherwise the peaks refer to components of fatty acids originating from lipids; diff., differences of the means values.

A limitation of this study is, that the patient group and the control group are not totally age-matched, so an effect of the age on the results may be possible. However, in literature age-dependend effects on plasma phospholipids are very small. Babin *et al*. found no significant alterations (29) whereas de Groot *et al*. found a small dependence on age (30). But, main effects on PL were due to the nutrition. Nevertheless, a point that should be taken into consideration when comparing the male collectives is that the male patients are older than the male volunteers. Therefore further investigations seem to be necessary.

Comparing the concentrations of substances detected in blood plasma of women with RCC with those of men with RCC no significant differences can be seen. Tendentially the women show slightly higher concentrations for the components of lipids. The biggest differences between both groups can be found for glyceryl groups of lipids.

### *In vivo* ^1^H MR spectroscopy

**Figure 7:**
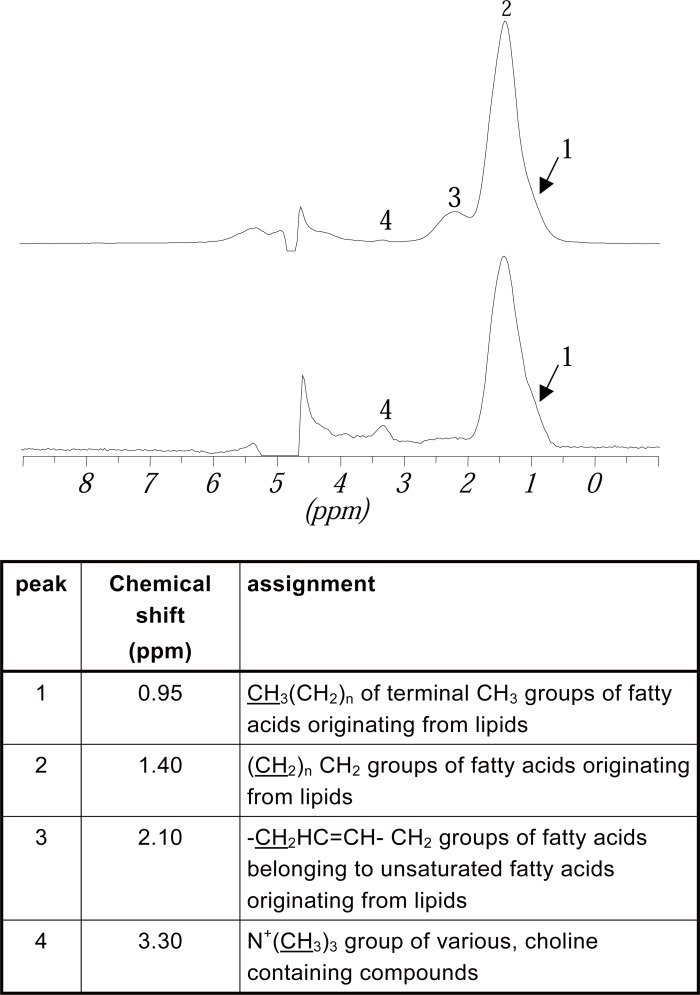
*In vivo* ^1^H MR spectra of the same part of the kidney. The upper spectrum was recorded with an echo time of 25 ms, the lower one with an echo time of 250 ms. The spectra have both been standardized on the same peak area (the one of Peak 2). Chemical shifts (in ppm) and assignment of the peaks has been made from higher to lower field.

The Figure 7 shows two *in vivo* ^1^H spectra, the upper one recorded with an echo time of 25 msec., the lower one with an echo time of 250 ms. The assignment of the peaks was made using literature data (24, 31, 32).

Peak 1 can be seen separately in some of the spectra but mostly is only a shoulder of the bigger signal at 1.40 ppm. Despite the peaks in Figure 7 in some spectra an additional peak at 2.88 ppm can be detected, which also belongs to unsaturated fatty acids from lipids. However, as this peak can not be seen in every spectrum it has not been evaluated in this investigation. The chemical shifts of the peaks can differ up to ± 0.04 ppm. However it is not possible to discriminate between small renal structures.

Comparing the spectra detected with an echo time of 25 ms with those detected with an echo time of 250 ms (Figure 7) it could be seen that the lipid signals which show a short T_2_ time are suppressed in spectra detected with a long T_2_ time whereas signals with a longer T_2_ time, like choline are emphasized. As the lipids are the object of interest in this investigation the spectra chosen for evaluation had been detected with a short echo time of 25 ms. This showed maximum intensity for the lipid signals and a good suppression of the water signal.

**Figure 8:**
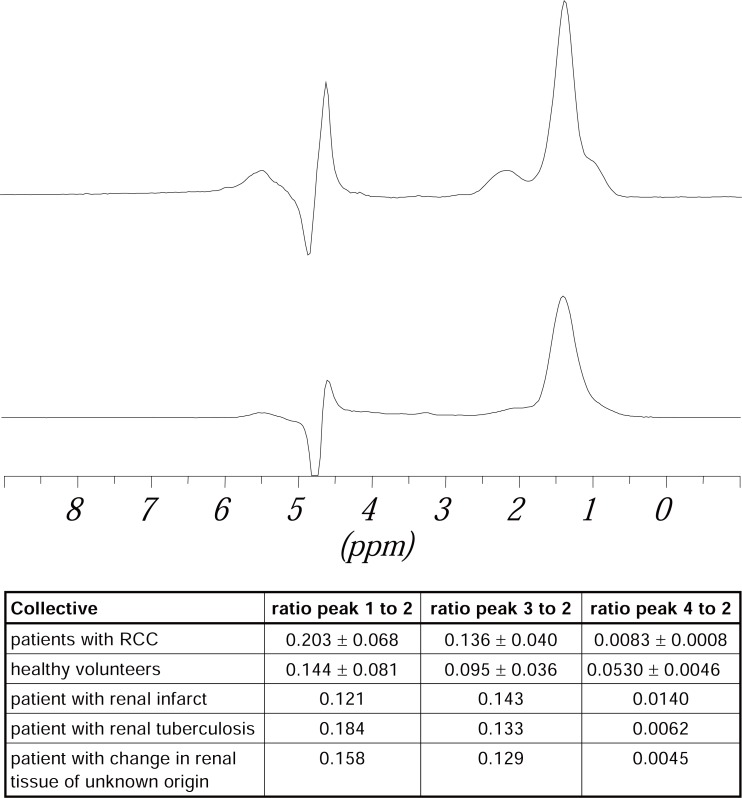
Spectrum of a patient with RCC (upper one) and spectrum of a healthy volunteer (lower one), recorded with an echo time of 25 ms in a volume of 20 × 20 × 20 mm. The assignment of the peaks is the same as in Figure 7. The (mean) values and standard deviations of the ratio of the peak areas (referred to the biggest signal) for the different collectives, determined from their spectra are listed in this table.

Figure 8 shows the spectrum and the values obtained for the peak areas (referenced to peak 2) of renal tumor tissue of a patient with RCC (upper one) and renal tissue of a healthy volunteer (lower one). Using *in-vivo* spectroscopy no addition of a reference substance was possible and as a consequence no quantitative determination of concentrations of the detected compounds. To make it possible to compare the spectra of patients and volunteers the peak areas were standardized to those of the biggest peak (peak 2).

Comparing spectra of tumor tissue fraom a patient with RCC to normal tissue of a healthy volunteer higher value for the ratio of the terminal CH_3_ groups of fatty acids (peak 1) to CH_2_ groups of fatty acids (peak 2) can be seen. The value of the ratio of CH_2_ groups of unsaturated fatty acids (peak 3) to CH_2_ groups of peak 2 is also higher for patients than volunteers. The value for the ratio of choline containing compounds (peak 4) to CH_2_ groups of fatty acids (peak 2) is lower for patients than volunteers. However, this lower value could be due to a higher content of lipids in the tumor tissue.

**Figure 9:**
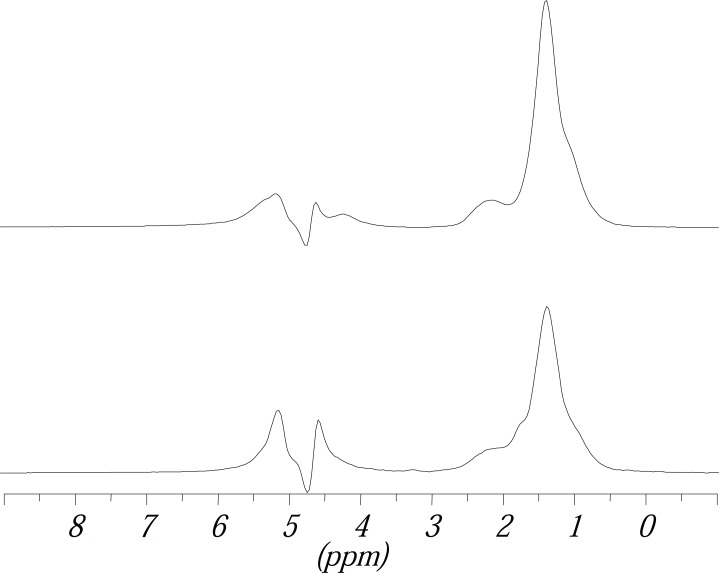
Two spectra of the same patient with RCC were image-guided using MR tomography. The upper spectrum belongs to tumor tissue, the lower one to healthy renal tissue of the same patient. The spectra were recorded with an echo time of 25 ms in a volume of 20 × 20 × 20 mm. The signals could be assigned in the same manner as the ones shown in Figure 7.

For the comparison of renal tumor with healthy renal tissue from the same patient the same results as reported above can be found (see also the Figure 9). However, it can be seen that the values for the ratios for lipid signals from healthy tissue of patients with RCC are higher than those obtained for healthy tissue from healthy volunteers (peak 1/peak 2 patients: 0.273, volunteers: 0.221, peak 3/peak 2 patients: 0.126, volunteers: 0.122). The value for the ratio of choline containing compounds (peak 4) to CH_2_ groups of fatty acids (peak 2) is lower for healthy renal tissue from patients than for healthy renal tissue from volunteers (patients: 0.0008, volunteers: 0.0275).

## DISCUSSION

### *In vitro* ^1^H MR spectroscopy of blood plasma

Comparing the concentrations of different components of lipids in blood plasma of patients with RCC with those obtained from healthy volunteers the patients generally show significant lower concentrations for components of fatty acids that can be assigned to CH_2_ groups of -CH_2_CH_2_COOR and -CH_2_CH_2_HC=CH-. Additionally the patients showed higher concentrations of lactate than the volunteers. The sum of the concentrations of fatty acids originating from VLDL and LDL and those of cholesterol from HDL didn’t differ significantly between patients and volunteers but still the concentrations were decreased in blood plasma of patients with RCC. However, other studies in patients with renal diseases (e.g. a nephrotic syndrome) show an increase of cholesterol, VLDL, LDL and triacylglycerines that means fatty acids containing molecules in blood plasma (33). That leads to the conclusion that the changes in the concentrations of components of lipids in blood plasma of patients with RCC are not due to a renal failure. In tissue from RCC itself an increased content of cholesterol, cholesterol esters, fatty acids (17, 34) and triacylglycerine (2) could be found in comparison to healthy renal tissue. The accumulation of these substances in tumor tissue, resulting from a higher need in the tumor, could lead to a general decrease of components of lipids in blood plasma.

### Patients with renal cell carcinoma, *in-vivo* ^1^H MR spectroscopy

Beside the investigation of blood plasma of the patients with RCC renal tumor tissue has been investigated using the *in vivo* ^1^H MR spectroscopy. The results were compared to those obtained from healthy renal tissue. In previously published studies using ^1^H-magic angle spinning MR spectroscopy we could show that renal tumor tissue displays multiple biochemical alterations, especially in the lipid metabolism (2, 16). The *in-vivo* spectra of tumor tissue now revealed also a higher value for the ratio of terminal CH_3_ groups to CH_2_ groups belonging to fatty acids when compared to renal tissue of healthy volunteers. For the ratio of CH_2_ groups originating from fatty acids to CH_2_ groups in general, the patients with RCC showed higher values too. In contrast to that the value for the ratio of choline containing compounds to CH_2_ groups of fatty acids was lower for patients than volunteers. These results were confirmed when healthy and tumor tissue was compared in the same patient. The peak assignments in the *in vivo* ^1^H MR spectra are not the same as the ones being made in a similar paper (26), but is in agreement with other investigators who investigated healthy renal tissue or blood plasma (25, 27, 33). The paper mentioned above did not contain any statements concerning the changes in signals of lipids in tumor tissue in comparison to healthy renal tissue.

The observation of higher values for lipids in tumor tissue in this examination is in good agreement with what can be seen in literature, investigations using electron microscopy showed lipid drops in cells of renal cell carcinoma (35).

## CONCLUSION

Renal cell carcinoma displays conspicuous alterations in cell lipid metabolism. This examinations show that in patients with RCC carcinoma tissue can be differentiated from normal renal tissue even by using the comparatively less resolutive *in-vivo* ^1^H MR spectroscopy in a whole body tomograph. Exceeding our expectations, we could demonstrate that pathobiochemical changes in the cancer metabolism are not limited to the tumor cells but also show a spread beyond. Corresponding systemic alterations which may cause e.g. paraneoplastic effects can also be detected using *in-vitro* ^1^H-HR-spectroscopy. Therefore, this work indicates the potential of ^1^H-MR spectroscopy for further examinations in tumor biology, diagnosis and eventually in therapy.
